# Serum uric acid levels and risk of cardiovascular disease in type 2 diabetes: results from a cross-sectional study and Mendelian randomization analysis

**DOI:** 10.3389/fendo.2023.1251451

**Published:** 2023-11-07

**Authors:** Ying He, Jincheng Feng, Bo Zhang, Qiong Wu, Yongjie Zhou, Diao He, Daofeng Zheng, Jiayin Yang

**Affiliations:** ^1^ Key Laboratory of Transplant Engineering and Immunology, Laboratory of Liver Transplantation, Frontiers Science Center for Disease-Related Molecular Network, West China Hospital, Sichuan University, Chengdu, Sichuan, China; ^2^ Department of Liver Transplantation, Union Hospital, Tongji Medical College, Huazhong University of Science and Technology, Wuhan, China; ^3^ Department of Critical Care Medicine, West China Hospital, Sichuan University, Chengdu, Sichuan, China; ^4^ Department of General Surgery and Liver Transplantation Center, West China Hospital, Sichuan University, Chengdu, Sichuan, China

**Keywords:** T2D, serum uric acid, cardiovascular disease, Mendelian randomization, heart failure

## Abstract

**Aims:**

Serum uric acid (SUA) levels have been previously linked to a higher risk of cardiovascular disease (CVD) in individuals with type 2 diabetes (T2D) according to various observational studies. However, whether this association is causally linked or simply influenced by confounding factors is unclear. Therefore, this study utilized Mendelian randomization (MR) analysis to explore the causality between SUA levels and the risk of CVD in individuals with T2D.

**Methods:**

Our study cohort consisted of 5723 participants who were diagnosed with T2D in the National Health and Nutrition Examination Survey (NHANES) from 1999-2018. The study assessed the association between SUA levels and the risk of CVD using a multivariable logistic regression model. To further examine causality between SUA levels and CVD, a two-sample MR study was conducted utilizing genetic data from genome-wide association studies (GWAS) involving over 140,000 individuals. The main MR analysis employed the inverse-variance-weighted (IVW) method. Additionally, several sensitivity analyses were performed to evaluate the robustness and pleiotropy of the results.

**Results:**

In the cross-sectional study, after multivariable adjustment, participants with SUA levels >6.7 mg/dL exhibited odds ratios (ORs) of 1.51 (95% CI: 1.01-2.26, p=0.049) for heart failure, 1.02 (95% CI: 0.69-1.50, p=0.937) for coronary heart disease, 1.36 (95% CI: 0.78-2.38, p=0.285) for angina, and 1.22 (95% CI: 0.80-1.85, p=0.355) for myocardial infarction when compared to participants with SUA levels ≤ 4.6 mg/dL. However, in the IVW analysis, no causality between SUA levels and the risk of heart failure was observed (OR = 1.03, 95% CI: 0.97-1.09, p = 0.293). The secondary analysis yielded similar results (OR = 1.05, 95% CI: 0.96-1.14, p = 0.299). The sensitivity analyses further supported our primary findings.

**Conclusion:**

Based on the MR study, we did not find supporting evidence for a causal association between SUA levels and the risk of heart failure.

## 1 Introduction

Diabetes mellitus affects approximately one in every 10.5 adults globally, with an estimated 90% of cases being T2D. This makes it a critical public health challenge of the 21st century (1, 2). T2D is typically associated with metabolic disorders, progressive insulin resistance and hyperglycemia (3). Prolonged hyperglycemia significantly increases the risk of microvascular and macrovascular complications, potentially resulting in premature mortality or disability (4, 5). Cardiovascular complications account for the most frequently occurring adverse events and remain the primary cause of mortality in this population (6). Up to 50% of patients may succumb to such complications, underscoring the need for robust prevention of CVD in T2D (7). Unfortunately, the prevention and treatment of CVD among individuals with diabetes may not present an optimistic outlook. Some large prospective cohort studies suggest that tight glucose control may not effectively mitigate the risk of cardiovascular complications and mortality (8). Even some hypoglycemic drugs may increase the risk of CVD (9). Therefore, it is crucial to identify effective intervention factors that can significantly reduce the risk of CVD. Uric acid, which remains a highly debated variable, has captured the attention of researchers.

Uric acid is considered a biologically inactive waste product resulting from purine metabolism, and it was identified as the cause of gout (10). Many observational studies have established a notable association between SUA levels and several cardiovascular conditions, leading to uric acid being recognized as a potential risk factor for CVD (11, 12). Nonetheless, the associations remain controversial. Some experts have contended that SUA may not be a substantial risk factor for CVD (13). One problem with determining the link between SUA and CVD is that elevated levels of SUA often coincide with identified cardiovascular risk factors, including hypertension, metabolic syndrome, and renal disease. Given that cardiovascular risk factors tend to be more prevalent in individuals with T2D, this population may be more susceptible to developing hyperuricemia due to hyperinsulinemia and chronic kidney disease (CKD) (14). There appears to be an accelerated progression of CVD in individuals with T2D, so it is particularly important to investigate the relationship between SUA levels and CVD risk in T2D. However, due to the limited research specifically addressing this issue in individuals with T2D, further studies are necessary to comprehensively understand the association between SUA levels and CVD in this specific group.

To assess the impact of SUA levels on CVD risk in individuals with T2D, we conducted an initial cross-sectional study and selected 5723 participants with T2D in the NHANES, which provides a large and nationally representative dataset that encompasses the U.S. population. Furthermore, we employed MR analysis to evaluate the causal correlation between SUA levels and the risk of CVD. MR is a statistical method that leverages genetic variants strongly associated with the exposure of interest to estimate a nonconfounded causal association between that exposure and an outcome (15). One of the key advantages of MR analysis is its ability to mitigate biases arising from reverse causality and uncontrolled confounders, since genetic variants are stable over time and are not susceptible to influence from environmental or other factors (16).

## 2 Materials and methods

### 2.1 Research design

The NHANES program is conducted in the U.S. by the National Center for Health Statistics of the Centers for Disease Control and Prevention. It originated in the early 1960s when a series of surveys were initiated to examine various health topics (17). In 1999, the survey transitioned into a continuous program, which adapted its focus to address emerging health and nutrition needs. An important characteristic of the NHANES program is its integration of both interviews and physical examinations. The interview component takes place in participants’ homes, followed by a standardized physical examination and laboratory tests (18). The comprehensive methodology for data collection and the data in our study are publicly available on the NHANES website (https://wwwn.cdc.gov/nchs/nhanes/default.aspx).

In this cohort study, we analyzed data from participants aged 18 or older with T2D from 10 cycles of NHANES from 1999-2018. Diabetes was defined based on self-reported physician diagnosis. Out of the initial 6699 participants who met the diagnostic criteria for diabetes, we excluded individuals who had type 1 diabetes (n=17) and excluded individuals who had missing data on SUA, BMI, HbA1c levels, and age at diagnosis (n=959). Ultimately, 5723 participants were included in our study.

### 2.2 Exposure serum uric acid measurement

SUA measurements were obtained from different laboratory instruments across multiple NHANES cycles. In NHANES 1999-2000, SUA was measured using a Hitachi Model 704 multichannel analyser. In NHANES 2001-2007, a Beckman Synchron LX20 instrument was used for SUA measurements. In NHANES 2008-2016, a Beckman UniCel DxC800 Synchron analyser was used for SUA measurements. Finally, in NHANES 2017-2018, SUA measurements were obtained using a Roche Cobas 6000 analyser. Previous studies have reported combining SUA data from multiple NHANES cycles for analysis (19, 20).

### 2.3 Assessment of covariates

Participants’ height (cm) and weight (kg) were measured to ascertain BMI. BMI is computed by dividing an individual’s weight in kilograms by the square of their height in meters. The classification is as follows: normal range (BMI<25 kg/m^2^), overweight (25 kg/m^2^<BMI<30 kg/m^2^), and obesity (BMI ≥ 30 kg/m^2^) (21). Hypertension is diagnosed when an individual’s systolic blood pressure is equal to or exceeds 140 mmHg and/or their diastolic blood pressure is equal to or exceeds 90 mmHg (22). Detailed laboratory methods for measuring HbA1c, lipid profiles, ALT, AST, creatinine, blood urea nitrogen (BUN), fasting insulin (FI), and fasting plasma glucose (FPG) are provided in the NHANES documentation. Insulin resistance was estimated using the homeostasis model assessment of insulin resistance (HOMA-IR), calculated by FI (μU/mL) * FPG (mmol/L)/22.5 (23).

Chronic kidney disease was determined by an estimated glomerular filtration rate of less than 60 mL/min/1.73 cm^2^ (24), calculated using the Chronic Kidney Disease Epidemiology Collaboration equation (25). Participants were categorized into three groups based on their smoking status: nonsmokers, former smokers, and current smokers. Educational level was calculated by the highest grade or the highest degree obtained. Physically active levels were determined using survey questionnaires. The physically active level was calculated by low-intensity, moderate-intensity or vigorous sports and fitness programs for more than 10 minutes per week (26).

### 2.4 Prescription medications

Participants were asked to provide information on any prescription medications they had taken in the 30 days leading up to the examination. Additionally, they were requested to bring their medication bottles to the examination for verification. Information regarding medications (uric acid-lowering medications and hypoglycemic medications) was then recorded accordingly.

### 2.5 Statistical analysis

All statistical analyses were performed using Rtools, version 4.2, taking into account the complex survey design of NHANES. We applied appropriate weighting for each analysis to ensure that our results accurately reflected the nationally representative estimates. Categorical variables are presented as numbers and weighted proportions, and continuous variables are presented as weighted means ± standard deviations (SDs). To examine the associations between variables, multivariable logistic regression models were employed to calculate ORs with 95% confidence intervals (95% CIs). Participants were categorized into four groups based on their SUA levels. To detect differences across the four groups, two different statistical tests were employed based on the variable type. Multivariable models were constructed to account for potential confounders. The proportional hazard assumption was assessed for all models to ensure that there were no violations. Stratified analyses were conducted within specific subgroups, including age, sex, race or ethnicity (white and nonwhite), duration of diabetes (<5 years, 5-10 years, ≥10 years), BMI (<25, 25-30, ≥30 kg/m^2^), hypertension (yes or no), smoking (never, ever, or current), alcohol intake in drinks per day (0, 1 to ≤2, >2), physical activity (low intensity, moderate intensity, high intensity), hyperlipidemia (yes or no), CKD (yes or no), HbA1c level (<7.0% or ≥7.0%), and diabetes treatment (insulin, insulin + oral medications, oral medications, no pharmacotherapy). Additionally, the study conducted several sensitivity analyses to assess the robustness of the main findings. First, to mitigate the potential impact of reverse causality, participants who were taking uric acid-lowering medications at the baseline examination were excluded. Second, the associations were evaluated after excluding individuals who were taking SGLT-2 inhibitors, as these medications have been shown to reduce cardiovascular risk (27). Third, participants with a history of CKD (eGFR<60 mL/min/1.73cm^2^) were excluded from the primary analyses. A p value < 0.05 (two-tailed) was considered statistically significant.

## 3 Mendelian randomization study

### 3.1 Data sources and study participants

Summary-level GWAS data for heart failure were obtained from two sources. The primary analysis was conducted by Sonia Shah et al. and included 47,309 heart failure cases and 930,014 controls. In this study, participants were diagnosed with heart failure without specific criteria based on left ventricular ejection fraction (LVEF), encompassing cases with both reduced and preserved heart function. Controls in this study comprised individuals without a diagnosis of heart failure (28). The secondary analysis used data from the FinnGen study, which comprised 19,676 heart failure cases and 272,371 controls of European descent. Heart failure diagnoses in the FinnGen data were based on the International Classification of Diseases code R7 (29). For more detailed information, the following link can be accessed: gs://finngen-public-data-r7/summary_stats/finngen_R7_I9_HEARTFAIL.gz.

### 3.2 Genetic instrument selection

Genetic instruments for SUA levels were obtained from a GWAS conducted by the Global Urate Genetics Consortium (GUGC). This GWAS included >140,000 individuals of European ancestry and identified and replicated genome-wide loci associated with SUA levels (30). The study further analyzed replicated and genome-wide significant uric acid-related SNPs in other populations. Specifically, this included 8,340 individuals of Indian descent, 5,820 African Americans, and 15,286 Japanese individuals. By examining these diverse populations, the study aimed to assess the broader applicability of the SNP associations beyond individuals of European ancestry.

### 3.3 Statistical analysis

To explore potential causal associations, we employed IVW of MR as our primary analysis (15). This method allowed us to estimate the effect of a 1-SD increase in SUA exposure on the risk of CVD. The IVW approach assumes no horizontal pleiotropy. To address the potential impact of horizontal pleiotropy, we conducted several sensitivity analyses using the MendelianRandomization package in R (31). These analyses included the weighted median, MREgger, simple mode, and Mendelian randomization pleiotropy RESidual sum and outlier (MR-PRESSO). In the IVW and MR−Egger analyses, we employed the “random” model to account for potential heterogeneity among the genetic instruments. Additionally, we utilized the “penalized” parameter to penalize variants with heterogeneous causal estimates (32). These sensitivity analyses helped to address potential biases arising from horizontal pleiotropy and provided additional insights into the robustness of our results.

## 4 Results

### 4.1 Characteristics of the participants at baseline

We identified 6699 participants who had been diagnosed with diabetes. Of these, 976 participants with missing SUA, BMI, HbA1c or age at diagnosis and participants with T1D were excluded. A total of 5723 participants were included in the study. The characteristics of the study sample are presented in **Table 1**. Participants had a mean age of 61.9 ( ± 13.2) years and were predominantly male (51.3%). Participants with the lowest SUA levels had a mean age of 56.4 ( ± 13.8) years, with a mean BMI of 30.8 ( ± 6.9) kg/m², and 60.0% were women. Among this group, 20.3% had retinopathy, 7.3% had chronic kidney disease, 7.7% had tumors, and 61.9% had hypertension. Among participants with the highest SUA levels, the mean age was 62.5 ( ± 12.9) years, the mean BMI was 34.8 ( ± 8.0) kg/m^2^, and 41.1% were women. Additionally, 22.7% of participants had retinopathy, 40.7% had chronic kidney disease, 11.7% had tumors, and 84.5% had hypertension, indicating the presence of higher diabetes comorbidities. Furthermore, participants with the lowest uric acid levels displayed higher levels of fasting glucose, HbA1c, estimated GFR, and HDL. Conversely, participants with the highest SUA levels exhibited higher levels of BUN, creatinine, and urinary albumin, suggesting the presence of renal dysfunction.

**Table 1:** Basic characteristics of participants according to uric acid status in NHANES 1999-2018.

Characteristics	Uric acid status				
	≤4.6 (n=1,525)	4.7-5.5 (n=1,388)	5.6-6.7 (n=1,471)	>6.7 (n=1,339)	*P*
Age, mean (SD)	56.4 (13.8)	59.9 (13.3)	60.1 (13.5)	62.5 (12.9)	<0.001
BMI, mean (SD)	30.8 (6.9)	32.0 (7.1)	33.6 (7.5)	34.8 (8.0)	<0.001
Gender, n (%)					
Male	595 (40.0)	694 (48.9)	845 (56.5)	801 (58.9)	<0.001
Female	930 (60.0)	694 (51.1)	626 (43.5)	538 (41.1)	
Weight status, kg/m^2^					
<25 kg/m^2^	309 (19.7)	228 (14.9)	161 (7.9)	120 (7.8)	<0.001
25 to <30 kg/m^2^	512 (30.6)	451 (28.7)	435 (26.5)	330 (22.4)	
≥30 kg/m^2^	704 (49.6)	709 (56.4)	875 (65.6)	889 (69.8)	
Race/ethnicity					
Mexican American	434 (12.3)	315 (9.6)	294 (9.2)	140 (4.2)	<0.001
Other Hispanic	176 (8.1)	129 (5.1)	128 (5.8)	83 (4.0)	
Non-Hispanic White	488 (59.7)	488 (62.4)	521 (61.9)	543 (64.7)	
Non-Hispanic Black	289 (12.1)	324 (13.5)	389 (15.2)	450 (18.1)	
Other Race	138 (7.8)	132 (9.5)	139 (7.9)	123 (9.0)	
Education level, n (%)					
High school or less	952 (50.5)	841 (53.1)	857 (49.2)	796 (50.4)	0.706
Some college	368 (31.2)	338 (27.7)	397 (32.5)	363 (30.8)	
College graduate	205 (18.3)	209 (19.2)	217 (18.3)	180 (18.7)	
Insurance, n (%)					
Any insurance	1264 (86.3)	1190 (88.9)	1303 (90.7)	1224 (92.0)	0.029
Uninsured	256 (13.3)	193 (10.7)	165 (9.2)	113 (7.7)	
Smoking status, n (%)					
Never	801 (50.9)	719 (50.0)	730 (49.2)	583 (44.9)	<0.001
Ever	434 (28.6)	448 (33.7)	514 (33.7)	579 (43.7)	
Current	290 (20.5)	221 (16.3)	227 (17.1)	177 (11.4)	
Alcohol, n (%)					
0 drinks/day	857 (49.5)	764 (47.6)	785 (47.8)	740 (50.2)	0.840
1-2 drinks/day	460 (36.5)	440 (39.6)	490 (38.4)	419 (35.9)	
>2 drinks/day	208 (14.1)	184 (12.8)	196 (13.9)	180 (13.8)	
Physical activity, n (%)					
Low intensity	786 (45.5)	713 (47.4)	779 (52.5)	764 (54.3)	0.014
Moderate-intensity	405 (28.6)	386 (29.0)	365 (24.3)	320 (24.8)	
High-intensity	334 (25.9)	289 (23.6)	327 (23.2)	255 (20.9)	
Comorbidities, n (%)					
Retinopathy	321 (20.3)	279 (18.1)	315 (19.4)	329 (22.7)	0.247
Chronic kidney disease	119 (7.3)	195 (12.4)	342 (19.8)	592 (40.7)	<0.001
Tumor	125 (7.7)	135(9.1)	121 (6.8)	161 (11.7)	0.004
Hypentension	991 (61.9)	979 (67.1)	1144 (76.7)	1129 (84.5)	<0.001
Diabetes mellitus treatment					<0.001
Insulin	229 (15.0)	140 (10.1)	169 (11.5)	198 (14.8)	
Insulin+oral medications	226 (14.8)	178 (12.8)	184 (12.5)	216 (16.1)	
Oral medications	826 (54.2)	839 (60.4)	890 (60.5)	753 (56.2)	
No pharmacotherapy	244 (16.0)	231 (16.6)	228 (15.5)	172 (12.8)	
Age at diagnosis, mean(SD)	46.5 (15.5)	50.1 (15.3)	50.6 (14.7)	51.9 (14.9)	0.122
Duration of diabetes, mean(SD)	12.2 (11.2)	11.7(11.8)	12.6 (12.2)	12.5 (12.3)	0.414
Biochemical profile, mean (SD)					
Glucose (mmol/L)	10.0 (4.3)	8.8 (3.5)	8.6 (3.4)	8.2 (3.1)	<0.001
HbA1c (%)	8.0 (2.1)	7.4 (1.7)	7.2 (1.7)	7.2 (1.5)	<0.001
HOMA-IR	9.0 (14.9)	7.2 (9.2)	10.1 (17.2)	10.3 (17.7)	<0.001
C-reactive protein (mg/dL)	2.1 (5.3)	2.6 (6.9)	2.4 (5.8)	3.6 (11.5)	0.009
ALT (U/I)	25.6 (20.0)	25.8 (26.2)	26.9 (19.0)	27.3 (41.8)	0.510
AST (U/I)	24.6 (19.5)	25.3 (23.5)	26.1 (14.2)	27.3 (23.3)	0.114
eGFR (ml/min/1.73 m^2^)	93.5 (22.4)	85.5 (22.7)	81.1 (24.2)	69.2 (26.5)	<0.001
Cholesterol (mmol/L)	4.9 (1.4)	4.8 (1.2)	4.8 (1.2)	4.7 (1.3)	0.245
Triglycerides (mmol/L)	2.2 (2.9)	2.3 (1.8)	2.4 (2.5)	2.4(2.1)	0.214
LDL-Cholesterol (mmol/L)	2.7 (1.0)	2.7 (0.9)	2.7 (1.0)	2.5 (0.9)	0.280
HDL-Cholesterol (mmol/L)	1.3 (0.4)	1.2 (0.4)	1.2(0.3)	1.2 (0.3)	<0.001
Blood urea nitrogen (mg/dl)	5.0 (2.1)	5.5 (2.2)	5.9 (2.5)	7.6 (3.8)	<0.001
Blood creatinine (mg/dl)	0.9 (0.7)	0.9 (0.7)	1.0 (0.7)	1.2 (0.6)	<0.001
Uric acid (mg/dl)	3.9 (0.6)	5.1 (0.3)	6.1 (0.3)	7.9 (1.1)	<0.001
Urinary albumin	82.7 (749.9)	88.5 (474.5)	152.5 (592.3)	268.1(964.8)	<0.001
Urine creatinine	104.3 (67.4)	111.93 (69.6)	120.67 (73.0)	121.4 (73.0)	<0.001

NHANES, National Health and Nutrition Examination Survey; ALT, Alamine aminotransferase; AST, Aspartate aminotransferase; eGFR, estimated glomerular filtration rate; HDL, high-density lipoprotein; LDL, low density lipoprotein; n, numbers of subjects; SD, standard deviation; %, weighted percentage.

### 4.2 Serum uric acid and cardiovascular disease

Without adjusting for variables, compared to participants with the lowest SUA levels, the ORs were 2.96 (95% CI, 2.09-4.18; P<0.001) for heart failure, 1.97 (95% CI, 1.46-2.65; P<0.001) for coronary heart disease, 2.04 (95% CI, 1.32-3.13; P=0.001) for angina/angina pectoris, and 1.92 (95% CI, 1.38-2.66; P<0.001) for myocardial infarction in the highest SUA levels. After adjusting for multiple variables, compared to participants with the lowest SUA levels, the ORs for the highest SUA levels were 1.51 (95% CI, 1.01-2.26; P=0.049) for heart failure, 1.02 (95% CI, 0.69-1.50; P=0.937) for coronary heart disease, 1.36 (95% CI, 0.78-2.38; P=0.285) for angina/angina pectoris, and 1.22 (95% CI, 0.80-1.85; P=0.355) for myocardial infarction (**Table 2**).

**Table 2:** OR (95% CIs) for cardiovascular disease according to uric acid status among participants in NHANES 1999-2018 (n=5,723).

	Group 1 (<4.6)	Group 2 (4.7-5.5)		Group 3 (5.6-6.7)		Group 4 (>6.7)	
	OR (95% CI)	OR (95% CI)	*P*	OR (95% CI)	*P*	OR (95% CI)	*P*
Nonadjusted							
Heart failure	1.00 (reference)	1.00 (0.69, 1.44)	0.999	1.37 (0.96, 1.96)	0.082	2.96 (2.09, 4.18)	<0.001
Coronary heart disease	1.00 (reference)	1.31 (0.91, 1.87)	0.144	1.47 (1.09, 1.99)	0.013	1.97 (1.46, 2.65)	<0.001
Angina/angina pectoris	1.00 (reference)	1.18 (0.76, 1.84)	0.450	1.41 (0.98, 2.04)	0.068	2.04 (1.32, 3.13)	0.001
Myocardial infarction	1.00 (reference)	1.16 (0.82, 1.63)	0.404	1.35 (0.97, 1.87)	0.080	1.92 (1.38, 2.66)	<0.001
Multivariable-adjusted ^a^							
Heart failure	1.00 (reference)	0.79 (0.53, 1.18)	0.249	0.95 (0.65, 1.41)	0.812	1.51 (1.01, 2.26)	0.049
Coronary heart disease	1.00 (reference)	1.03 (0.71, 1.51)	0.862	1.06 (0.76, 1.48)	0.715	1.02 (0.69, 1.50)	0.937
Angina/angina pectoris	1.00 (reference)	1.00 (0.60, 1.64)	0.992	1.11 (0.75, 1.64)	0.611	1.36 (0.78, 2.38)	0.285
Myocardial infarction	1.00 (reference)	0.99 (0.69, 1.43)	0.960	1.07 (0.74, 1.56)	0.705	1.22 (0.80, 1.85)	0.355

NHANES, National Health and Nutrition Examination Survey; OR, odds ratio; CI, confidence interval; n, the number.

a Data were adjusted for age, sex, race or ethnicity, education level, body mass index, smoking status, drinking status, hypentension, physical activity, total cholesterol, triglycerides, HbA1C, high-density lipoprotein, blood urea nitrogen, and blood creatinine.

The sensitivity analyses excluded participants who were diagnosed with gout and took uric acid-lowering medicines at the baseline examination (**Supplementary Table 1**). In addition, participants who took SGLT2 inhibitors at baseline (**Supplementary Table 2**) and participants with CKD (eGFR <60 mL/min/1.73 cm²) at baseline (**Supplementary Table 3**) were also excluded from the analysis. The results did not show substantial changes in the associations across the different sensitivity analysis models.

There was an interaction between SUA and race and HbA1c and the risk of heart failure (P <0.05 for interaction). In the subgroup analysis for non-Hispanic white individuals to the group 1 (<4.6 mg/dL), the ORs for heart failure were 0.76 (95% CI, 0.44-1.29) in group 3 (5.6-6.7 mg/dL) and 1.17 (95% CI, 0.69-1.97) in group 4 (>6.7 mg/dL). In the subgroup analysis involving other racial groups compared to group 1 (<4.6 mg/dL), the ORs for heart failure were 1.73 (95% CI, 1.13-2.65) in group 3 (5.6-6.7 mg/dL) and 2.86 (95% CI, 1.72-4.77) in group 4 (>6.7 mg/dL). In the subgroup analysis of participants with HbA1c <7.0%, compared to group 1 (<4.6 mg/dL), the ORs for heart failure were 1.14 (95% CI, 0.80-1.62) in group 3 (5.6-6.7 mg/dL) and 2.17 (95% CI, 1.57-3.03) in group 4 (>6.7 mg/dL). Additionally, in the subgroup analysis of participants with HbA1c ≥ 7.0%, compared to group 1 (<4.6 mg/dL), the ORs for heart failure were 2.00 (95% CI, 1.38-2.91) in group 3 (5.6-6.7 mg/dL) and 4.18 (95% CI, 2.98-5.94) in group 4 (>6.7 mg/dL). No significant interactions were observed between sex, age, BMI, hypertension, smoking, alcohol consumption, physical activity, or CKD categories and the risk of heart failure (**Table 3**).

**Table 3:** Stratified analyses of the associations (OR, 95% CIs) between uric acid status and cardiovascular disease among participants in NHANES 1999-2018 (n=5,723).

	Group 1 (<4.6)	Group 2 (4.7-5.5)	Group 3 (5.6-6.7)	Group 4 (>6.7)	*P* for interaction
	OR (95% CI) ^a^	OR (95% CI) ^a^	OR (95% CI) ^a^	OR (95% CI) ^a^	
Age, years					0.291
<60	1.00 (reference)	0.87 (0.40, 1.92)	1.37 (0.72, 2.62)	1.74 (0.73, 4.15)	
≥60	1.00 (reference)	0.76 (0.48, 1.19)	0.84 (0.52, 1.37)	1.41 (0.91, 2.20)	
Gender					0.725
Male	1.00 (reference)	0.70 (0.39, 1.25)	0.85 (0.51, 1.44)	1.4 (0.81, 2.41)	
Female	1.00 (reference)	0.84 (0.48, 1.46)	0.96 (0.52, 1.77)	1.43 (0.8, 2.56)	
Race					0.034
Non-Hispanic White	1.00 (reference)	0.69 (0.40, 1.18)	0.76 (0.44, 1.29)	1.17 (0.69, 1.97)	
Other Race	1.00 (reference)	1.12 (0.67, 1.88)	1.73 (1.13, 2.65)	2.86 (1.72, 4.77)	
Weight status, kg/m^2^					0.536
<25	1.00 (reference)	1.95 (0.71, 5.32)	1.28 (0.33, 4.94)	2.41 (0.59, 9.91)	
25 to <30	1.00 (reference)	0.68 (0.35, 1.34)	0.85 (0.45, 1.62)	1.25 (0.64, 2.43)	
≥30	1.00 (reference)	0.69 (0.39, 1.22)	0.97 (0.57, 1.64)	1.45 (0.85, 2.49)	
Hypertension					0.099
Yes	1.00 (reference)	3.69 (1.40, 9.75)	3.21 (1.20, 8.54)	6.98 (2.45, 19.87)	
No	1.00 (reference)	0.63 (0.41, 0.98)	0.79 (0.52, 1.19)	1.18 (0.76, 1.84)	
Smoking status					0.883
Never	1.00 (reference)	0.84 (0.46, 1.55)	1.04 (0.55, 1.94)	1.94 (1.08, 3.48)	
Ever	1.00 (reference)	0.85 (0.44, 1.66)	1.06 (0.55, 2.07)	1.12 (0.59, 2.12)	
Current	1.00 (reference)	0.63 (0.27, 1.50)	0.65 (0.29, 1.47)	2.36 (0.92, 6.02)	
Physical activity					0.675
Low intensity	1.00 (reference)	0.94 (0.59, 1.50)	1.08 (0.73, 1.60)	1.50 (0.96, 2.35)	
Moderate intensity	1.00 (reference)	0.59 (0.23, 1.55)	0.99 (0.39, 2.53)	1.28 (0.45, 3.66)	
High intensity	1.00 (reference)	0.54 (0.20, 1.49)	0.59 (0.22, 1.56)	1.66 (0.61, 4.55)	
Duration of diabetes, year					0.656
<5Y	1.00 (reference)	0.86 (0.46, 1.58)	1.45 (0.85, 2.52)	2.66 (1.63, 4.48)	
5-10Y	1.00 (reference)	1.49 (0.77, 2.96)	1.77 (0.94, 3.43)	3.89 (2.19, 7.24)	
≥10Y	1.00 (reference)	0.95 (0.65, 1.37)	1.58 (1.13, 2.20)	3.22 (2.38, 4.41)	
HbA1c, %					0.011
< 7.0	1.00 (reference)	0.83 (0.57, 1.23)	1.14 (0.80, 1.62)	2.17 (1.57, 3.03)	
≥ 7.0	1.00 (reference)	1.11 (0.73, 1.69)	2.00 (1.38, 2.91)	4.18 (2.98, 5.94)	
Diabetes treatment					0.411
Insulin	1.00 (reference)	1.00 (0.50, 1.92)	2.03 (1.17, 3.57)	3.78 (2.30, 6.36)	
Insulin+oral medications	1.00 (reference)	1.38 (0.75, 2.52)	1.20 (0.65, 2.22)	2.47 (1.47, 4.26)	
Oral medications	1.00 (reference)	0.90 (0.58, 1.37)	1.77 (1.23, 2.58)	2.96 (2.09, 4.26)	
No pharmacotherapy	1.00 (reference)	1.42 (0.67, 3.05)	1.16 (0.53, 2.56)	4.22 (2.19, 8.58)	
Alcohol (drinks/day)					0.281
0	1.00 (reference)	0.84 (0.56, 1.26)	1.36 (0.91, 2.04)	1.81 (1.14, 2.86)	
1 to ≤2	1.00 (reference)	0.66 (0.30, 1.46)	0.52 (0.22, 1.22)	1.28 (0.58, 2.79)	
>2	1.00 (reference)	0.97 (0.23, 4.17)	0.65 (0.19, 2.23)	0.71 (0.19, 2.64)	
Chronic kidney disease					0.093
Yes	1.00 (reference)	2.08 (1.09, 3.96)	1.23 (0.65, 2.32)	1.69 (0.93, 3.07)	
No	1.00 (reference)	0.54 (0.33, 0.88)	0.84 (0.52, 1.36)	1.48 (0.88, 2.47)	

NHANES, National Health and Nutrition Examination Survey; OR, odds ratio; CI, confidence interval; n, the number.

a Data were adjusted for age, sex, race or ethnicity, education level, body mass index, smoking status, drinking status, hypentension, physical activity, total cholesterol, triglycerides, HbA1C, high-density lipoprotein, blood urea nitrogen, and blood creatinine.

### 4.3 Mendelian randomization study

Following application of the selection criteria for SNPs, we identified a total of 20 SNPs from the Shah et al. dataset and 44 SNPs from the FinnGen dataset that met the criteria for analyzing the effects of SUA on heart failure. Detailed descriptions of the selected SNPs can be found in **Supplemental Tables 4**, **5**.

In the primary analysis using the IVW model, there was no suggestion of a causal association between a 1-SD change in levels of SUA and the risk of heart failure (OR: 1.05, 95% CI: 0.97-1.14, p=0.241). The sensitivity analyses utilizing different methods, including weighted median (OR: 0.97, 95% CI: 0.91~1.03, p=0.332), simple mode (OR: 1.24, 95% CI: 1.00~1.53, p=0.058), and weighted mode (OR: 0.96, 95% CI: 0.91~1.02, p=0.232), provided consistent results. The MR−Egger regression test showed the presence of some unbalanced horizontal pleiotropy (P intercept =0.002). The Q test revealed no evidence of heterogeneity in the effect of SUA variants on the risk of heart failure (P Cochran’s Q =0.784). We confirmed this causality between SUA and the risk of heart failure by utilizing the FinnGen database, which yielded comparable results (OR: 1.05, 95% CI: 0.96-1.14, p=0.299) (**Table 4**).

**Table 4:** MR results for association between serum urate concentrations and heart failure.

Exposure	Outcome	No.SNP	Methods	OR	95%CI	*P*	Horizontal pleiotropy *P*_for Egger intercept_	Heterogeneity *P*_for Cochran’s Q_	*P*_for MR PRESSO global test_
Urate	Heart failure/GWAS	20	IVW	1.03	0.97-1.09	0.293		0.784	
			WM	0.97	0.91-1.03	0.332			
			MR−Egger	0.90	0.82-0.98	0.023	0.002		
			Simple mode	1.24	1.00-1.53	0.058			
			Weighted mode	0.96	0.91-1.02	0.232			
			MR-PRESSO	1.08	1.00-1.17	0.079			0.072
	Heart failure/FinnGen	44	IVW	1.05	0.96-1.14	0.299		0.205	
			WM	1.04	0.94-1.17	0.432			
			MR−Egger	0.95	0.81-1.11	0.509	0.136		
			Simple mode	1.08	0.97-1.21	0.057			
			Weighted mode	1.05	0.96-1.02	0.302			
			MR-PRESSO	1.05	0.96-1.14	0.305			0.591

MR, mendelian randomization; IVW, inverse-variance weighted, WM, weighted median; MR-PRESSO, MR pleiotropy residual sum and outlier.

**Figure 1:**
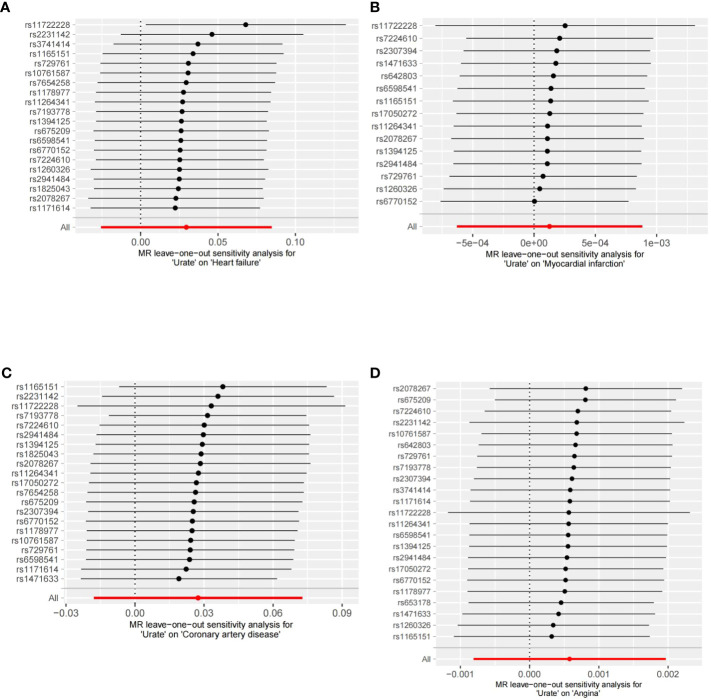
Leave-one-out analyses of the association between SUA traits and CVD. **(A)** Leave-one-out analyses of the association between SUA traits and heart failure. **(B)** Leave-one-out analyses of the association between SUA traits and myocardial infarction. **(C)** Leave-one-out analyses of the association between SUA traits and coronary artery disease. **(D)** Leave-one-out analyses of the association between SUA traits and angina.

In our analysis, we did not find a causal relationship between SUA and the risk of coronary artery disease (OR: 1.03, 95% CI: 0.98-1.08, p=0.235), angina (OR: 1.00, 95% CI: 0.99-1.01, p=0.414), or myocardial infarction (OR: 1.00, 95% CI: 0.99-1.01, p=0.744). The consistency of these results across different methods was demonstrated and can be seen in **Supplementary Table 6**. The leave-one-out analyses, depicted in **Figure 1**, did not identify any single nucleotide polymorphisms (SNPs) that strongly influenced the estimates. Additionally, the results from the forest scatter plots (**Supplement Figure S1**) and scatter plots (**Supplement Figure S2**) further support the findings from the main analysis, showing no causal relationship between SUA levels and these cardiovascular outcomes.

## 5 Discussion

This study employed a cross-sectional study utilizing data from the NHANES. Additionally, a two-sample MR analysis with summary data from GWAS was conducted to explore the potential association between SUA levels and the risks of CVD in individuals with T2D. Our observational research findings revealed that higher SUA levels were associated with an increased risk of heart failure. However, no significant associations were observed between SUA levels and coronary heart disease, angina pectoris, or myocardial infarction. Nevertheless, the MR analysis indicated that the association between SUA levels and heart failure may not be causal.

Although numerous studies have explored the relationship between SUA levels and the risk of CVD, a definitive conclusion has yet to be reached. Some epidemiological studies have analyzed the link between SUA levels and CVD, revealing suggestive evidence of an association (33, 34). However, a systematic review examining the existing evidence on the associations between SUA levels and several health outcomes found that the evidence for a definitive role of SUA levels in CVD outcomes was not compelling. While there was highly suggestive evidence for associations between SUA levels and CVD, it is important to acknowledge that a significant proportion of the meta-analyses exhibited substantial heterogeneity (I^2^ > 50%). This suggests that caution is needed when interpreting these associations (11).

Although observational studies cannot establish a causality between SUA levels and CVD in individuals, the use of uric acid-lowering medications, such as oxypurinol and allopurinol, in patients with symptomatic heart failure has also generated conflicting findings. For example, one large RCT did not find any improvements in clinical outcomes with oxypurinol, and the EXACT-HF trial found no significant differences in clinical status, heart failure scores, or 6-minute walk distances between patients with symptomatic heart failure who received allopurinol or placebo, despite reductions in SUA levels with allopurinol (35, 36). However, two small double-blind placebo-controlled RCTs reported potential benefits of allopurinol in improving endothelial function in patients with heart failure (37). Furthermore, an additional small RCT suggested that the beneficial effect of allopurinol on endothelial function in patients with heart failure may be dependent on the dosage administered (38). Overall, it is important to note that the current body of research has not yet provided conclusive evidence on the impact of SUA levels on CVD. Further investigation is necessary to gain a comprehensive understanding of the potential relationship between SUA levels and CVD in the general or diabetes population.

Compared to the general population, individuals with diabetes exhibit specific characteristics. In our study, we identified a notable observed relationship between elevated SUA levels and an increased risk of heart failure, coronary heart disease, angina, and myocardial infarction in individuals with T2D without adjusting for variables. However, once we accounted for multiple confounding variables, the previously observed association between SUA levels and CVD became less conclusive, and only heart failure remained significantly correlated with SUA levels. These findings highlight the importance of accounting for potential confounding factors when examining the relationship between SUA levels and CVD outcomes. In individuals with T2D, hypertension and CKD may serve as relevant confounding factors that can contribute to elevated SUA levels. Considering these factors is essential in accurately assessing the impact of SUA levels on CVD outcomes in individuals with T2D and increasing the risk of CVD and other adverse outcomes. In our study, only 7.3% of T2D patients with the lowest SUA levels had chronic kidney disease, and 61.9% had hypertension. However, at the highest SUA levels, 40.7% of T2D patients had chronic kidney disease, and 84.5% had hypertension.

However, our subgroup analysis revealed an interaction between SUA and race and HbA1c. Specifically, among non-Hispanic white individuals, increasing SUA levels were not associated with the risk of heart failure, whereas other racial groups experienced an elevated risk of heart failure as SUA increased. In the subgroup with HbA1c<7.0%, the risk of heart failure only showed an increase in the highest SUA group. However, in the subgroup with HbA1c>7.0%, the risk of heart failure was elevated even at lower uric acid concentrations. Although age, sex, hypertension, and chronic kidney disease are known to influence uric acid levels in the general or diabetes population, research examining specific subgroups of individuals with diabetes suggests that these factors may not have an impact on the interaction between uric acid and heart failure risk. These findings suggest that race and HbA1c may be important factors to consider in the assessment of the relationship between SUA and heart failure risk in the U.S. diabetes population.

One potential explanation for the observed association between SUA levels and heart failure is the possibility of residual confounding. There may be other factors besides SUA that contribute to heart failure risk, such as dietary factors, hypoglycemic medications, duration of diabetes or other comorbidities. To mitigate the effects of confounding factors, a two-sample MR analysis was performed to evaluate the potential causal relationship between SUA levels and CVD. After conducting both primary and sensitivity MR analyses, we found no evidence to support a causal relationship between genetically predicted SUA levels and CVD. However, our observational study did suggest a potential association between uric acid levels and heart failure. Our finding aligns with a few studies that have shown a lack of evidence linking increased SUA levels directly to heart failure in patients with T2D. Furthermore, this study contributes to the literature on this subject by using genetic variations associated with SUA levels as instrumental variables, which provides stronger evidence for a lack of causal relationship.

The current study possesses several notable strengths, including the use of a large sample size with multiple CVD outcomes and adjustment for relevant confounders. Additionally, the Mendelian randomization analysis provided robust evidence of the causality between SUA levels and heart failure risk. However, there are several limitations within the scope of the current study. To enhance the statistical power and broaden the scope of our study, we combined NHANES data, but our study might still lack sufficient statistical power to detect small or subtle effects. Additionally, we recognize that some unmeasured or inadequate variables might have differed between groups and could have influenced the observed outcomes. Furthermore, the possibility of T1D cannot be entirely excluded from our study. The NHANES database has provided explicit diagnoses of T1D and T2D since 2013. There are limitations to Mendelian randomization analyses, as they were not able to completely rule out the pleiotropy of SNP levels. Urate transporters are mostly associated with UA underexcretion than UA hyperproduction (such as URAT1, SLC2A9, GLUT9) (39). Therefore, the variability and diversity of urate transporters may have different effects on uric acid concentration. However, in this study, we did not examine relevant SNPs related to urate transporters. This limitation emphasizes the importance of future research in exploring the association between genetic variations in urate transporters and uric acid concentration. Additionally, there can be differences in SNPs among different racial and ethnic groups. These genetic variations can differ among various ethnic and geographic groups due to differences in population history, migration patterns, and genetic admixture. Therefore, it is important to consider these potential differences when studying the impact of SNPs on health outcomes in different populations. Mendelian analysis is based on the genetic pattern of a single gene, considering its influence on a specific trait (40). However, many traits are determined by multiple genes and environmental factors, neglecting these complexities. Mendelian analysis primarily focuses on the direct impact of genotypes on traits, overlooking the role of gene expression and regulation. In fact, the same genotype may exhibit different phenotypes in different individuals, which is associated with gene expression under different environmental conditions.

In conclusion, the current study provides evidence to suggest that elevated SUA levels may increase the risk of heart failure in individuals with T2D and related comorbidities but not necessarily other types of CVD. However, the MR analysis did not support a causal relationship between SUA levels and heart failure risk, indicating that other factors may play a causal role in heart failure development among those with T2D. Further research is needed to identify these factors and investigate their potential interactions with SUA levels.

## Data availability statement

The original contributions presented in the study are included in the article/**Supplementary Material**. Further inquiries can be directed to the corresponding author.

## Ethics statement

The survey protocols of the NHANES were approved by the Research Ethics Review Board of the National Center for Health Statistics (https://www.cdc.gov/nchs/nhanes/irba98.htm), and all survey participants provided informed consent.

## Author contributions

YH and JF had full access to all of the data in the study, takes responsibility for the integrity of the data and the accuracy of the data analysis, and wrote the paper. YH and JF contributed equally to this work. QW, BZ and YZ: provided continuous support and conceptual advice for this study. DH and DZ: analyzed the data and discussed the results. JY: designed the research, provided continuous support and conceptual advice for this study, is the guarantor of this work, had full access to all the data in the study and takes responsibility for the integrity of the data and the accuracy of the data analysis. Supervision: JY. All authors contributed to the articles and approved the submitted version.
