# Supplementary material for: Serum uric acid levels and risk of cardiovascular disease in type 2 diabetes: results from a cross-sectional study and Mendelian randomization analysis
Source: Front Endocrinol (Lausanne). 2023 Nov 7;14:1251451. doi: 10.3389/fendo.2023.1251451 (PMC10664243; doi:10.3389/fendo.2023.1251451)
Supplement: Supplementary file 1 [file DataSheet_1.pdf]

Supplement Table 1 OR (95% CIs) for cardiovascular disease according to uric acid status among participants after excluding subjects who were taking urico-lowering medications (n=5,493)

	Group 1 (≤4.6)	Group 2 (4.7-5.5)		Group 3 (5.6-6.7)		Group 4 (>6.7)	
	OR (95% CI)	OR (95% CI)	*P*	OR (95% CI)	*P*	OR (95% CI)	*P*
Non-adjusted							
Heart failure	1.00 (reference)	0.95 (0.64, 1.39)	0.777	1.42 (0.98, 2.06)	0.069	3.05 (2.13, 4.37)	<0.001
Coronary heart disease	1.00 (reference)	1.30 (0.89, 1.90)	0.177	1.52 (1.10, 2.09)	0.012	2.03 (1.47, 2.79)	<0.001
Angina/angina pectoris	1.00 (reference)	1.22 (0.77, 1.96)	0.399	1.45 (1.00, 2.11)	0.053	2.09 (1.33, 3.31)	0.002
Myocardial infarction	1.00 (reference)	1.22 (0.85, 1.76)	0.289	1.41 (0.99, 2.00)	0.057	1.96 (1.38, 2.79)	<0.001
Multivariable-adjusted [a]							
Heart failure	1.00 (reference)	0.74 (0.49, 1.12)	0.159	1.01 (0.66, 1.53)	0.971	**1.64 (1.05, 2.56)**	**0.031**
Coronary heart disease	1.00 (reference)	1.01 (0.68, 1.52)	0.946	1.09 (0.76, 1.56)	0.652	1.05 (0.68, 1.62)	0.826
Angina/angina pectoris	1.00 (reference)	1.02 (0.59, 1.76)	0.937	1.13 (0.74, 1.72)	0.564	1.40 (0.75, 2.63)	0.290
Myocardial infarction	1.00 (reference)	1.04 (0.70, 1.55)	0.842	1.14 (0.76, 1.71)	0.535	1.30 (0.81, 2.08)	0.285

NHANES, National Health and Nutrition Examination Survey; OR, odds ratio; CI, confidence interval; n, the number;

[a] Data were adjusted for age, sex, race or ethnicity, education level, body mass index, smoking status, drinking status, hypentension, physical activity, total cholesterol, triglycerides, HbA1C, high-density lipoprotein, blood urea nitrogen, and blood creatinine.

Supplement Table 2 OR (95% CIs) for cardiovascular disease according to uric acid status among participants after excluding subjects who were taking hypoglycemic medications (n=5,595)

	Group 1 (≤4.6)	Group 2 (4.7-5.5)		Group 3 (5.6-6.7)		Group 4 (>6.7)	
	OR (95% CI)	OR (95% CI)	*P*	OR (95% CI)	*P*	OR (95% CI)	*P*
Non-adjusted							
Heart failure	1.00 (reference)	1.02 (0.71, 1.46)	0.935	1.42 (1.00, 2.01)	0.054	3.00 (2.13, 4.23)	<0.001
Coronary heart disease	1.00 (reference)	1.34 (0.91, 1.97)	0.134	1.44 (1.06, 1.95)	0.020	1.95 (1.43, 2.66)	<0.001
Angina/angina pectoris	1.00 (reference)	1.17 (0.75, 1.84)	0.484	1.41 (0.99, 2.00)	0.058	2.06 (1.34, 3.17)	0.001
Myocardial infarction	1.00 (reference)	1.16 (0.82, 1.65)	0.410	1.38 (1.00, 1.89)	0.049	2.01 (1.46, 2.77)	<0.001
Multivariable-adjusted [a]							
Heart failure	1.00 (reference)	0.82 (0.55, 1.22)	0.333	1.02 (0.69, 1.50)	0.933	**1.57 (1.05, 2.34)**	**0.029**
Coronary heart disease	1.00 (reference)	1.08 (0.72, 1.62)	0.708	1.07 (0.77, 1.48)	0.708	1.03 (0.70, 1.53)	0.865
Angina/angina pectoris	1.00 (reference)	1.01 (0.61, 1.69)	0.961	1.14 (0.78, 1.66)	0.508	1.44 (0.82, 2.54)	0.203
Myocardial infarction	1.00 (reference)	1.02 (0.71, 1.48)	0.906	1.15 (0.81, 1.63)	0.442	1.37 (0.92, 2.02)	0.120

NHANES, National Health and Nutrition Examination Survey; OR, odds ratio; CI, confidence interval; n, the number;

[a] Data were adjusted for age, sex, race or ethnicity, education level, body mass index, smoking status, drinking status, hypentension, physical activity, total cholesterol, triglycerides, HbA1C, high-density lipoprotein, blood urea nitrogen, and blood creatinine.

Supplement Table 3 OR (95% CIs) for cardiovascular disease according to uric acid status among participants after excluding patients with chronic kidney disease (n=4,475)

	Group 1 (≤4.6)	Group 2 (4.7-5.5)		Group 3 (5.6-6.7)		Group 4 (>6.7)	
	OR (95% CI)	OR (95% CI)	*P*	OR (95% CI)	*P*	OR (95% CI)	*P*
Non-adjusted							
Heart failure	1.00 (reference)	0.66 (0.42, 1.04)	0.075	1.13 (0.74, 1.75)	0.573	2.07 (1.31, 3.25)	0.002
Coronary heart disease	1.00 (reference)	1.25 (0.84, 1.87)	0.267	1.27 (0.90, 1.80)	0.172	1.30 (0.86, 1.97)	0.217
Angina/angina pectoris	1.00 (reference)	1.05 (0.66, 1.67)	0.831	1.15 (0.76, 1.74)	0.508	1.70 (1.01, 2.87)	0.047
Myocardial infarction	1.00 (reference)	1.20 (0.81, 1.79)	0.361	1.16 (0.78, 1.73)	0.465	1.67 (1.13, 2.49)	0.012
Multivariable-adjusted [a]							
Heart failure	1.00 (reference)	0.54 (0.33, 0.88)	0.014	0.84 (0.52, 1.36)	0.480	**1.48 (1.08, 2.47)**	**0.038**
Coronary heart disease	1.00 (reference)	0.98 (0.64, 1.52)	0.940	0.88 (0.59, 1.32)	0.541	0.77 (0.46, 1.28)	0.310
Angina/angina pectoris	1.00 (reference)	0.88 (0.51, 1.52)	0.642	0.86 (0.53, 1.39)	0.537	1.17 (0.60, 2.30)	0.648
Myocardial infarction	1.00 (reference)	1.07 (0.70, 1.64)	0.751	0.93 (0.58, 1.49)	0.769	1.28 (0.76, 2.16)	0.362

NHANES, National Health and Nutrition Examination Survey; OR, odds ratio; CI, confidence interval; n, the number;

[a] Data were adjusted for age, sex, race or ethnicity, education level, body mass index, smoking status, drinking status, hypentension, physical activity, total cholesterol, triglycerides, HbA1C, high-density lipoprotein, blood urea nitrogen, and blood creatinine.

Supplement Table 4 Association of Urate-Associated SNPs with heart failure in GWAS database

SNP	closest gene	Effect allele (A1)	Other allele (A2)	SUA			heart failure		
				effect (mg/dl)	s.e.	p-value	effect (mg/dl)	s.e.	p-value
rs2231142	ABCG2	t	g	0.217	0.006	1.00E-134	-0.0274	0.0247	0.267
rs17050272	INHBB	a	g	0.037	0.006	9.40E-09	-0.005	0.0088	0.5691
rs6598541	IGF1R	a	g	0.044	0.006	5.20E-13	-0.0142	0.0151	0.3485
rs6770152	SFMBT1	t	g	-0.048	0.006	2.70E-16	0.0167	0.0167	0.3164
rs11264341	TRIM46	t	c	-0.048	0.006	1.00E-14	0.0254	0.0204	0.2132
rs1260326	GCKR	t	c	0.077	0.006	1.30E-40	0.014	0.0098	0.1525
rs675209	RREB1	t	c	0.063	0.006	1.40E-21	0.0068	0.0085	0.4248
rs11264341	TRIM46	t	c	-0.048	0.006	1E-14	-0.0259	0.0084	0.08277
rs1260326	GCKR	t	c	0.077	0.006	1.3E-40	0.0184	0.0081	0.02253
rs675209	RREB1	t	c	0.063	0.006	1.4E-21	0.0008	0.0085	0.9281
rs1165151	SLC17A1	t	g	-0.093	0.005	4.5E-60	0.0117	0.0097	0.2285
rs729761	VEGFA	t	g	-0.046	0.006	3.1E-12	-0.0186	0.0169	0.2702
rs1178977	BAZ1B	a	g	0.05	0.007	6.7E-12	0.0011	0.0115	0.9224
rs2941484	HNF4G	t	c	0.049	0.006	3.9E-17	-0.0114	0.0122	0.3516
rs1171614	SLC16A9	t	c	-0.074	0.007	6.5E-23	-0.0178	0.008	0.02675
rs2078267	SLC22A11	t	c	-0.078	0.006	8.7E-36	0.0118	0.0079	0.1343
rs3741414	INHBC	t	c	-0.071	0.007	9.8E-22	0.0136	0.0079	0.002168
rs1394125	UBE2Q2	a	g	0.043	0.006	9.8E-11	0.0069	0.0083	0.4034
rs6598541	IGF1R	a	g	0.044	0.006	5.2E-13	0.0066	0.0078	0.3966
rs7224610	HLF	a	c	-0.038	0.006	4.7E-11	0.0409	0.008	3.318E-07

Abbreviations: SNP, single nucleotide polymorphism; SUA, serum uric acid; GWAS, genome-wide association study; s.e.,standard error

Supplement Table 5   Association of Urate-Associated SNPs with heart failure in FinnGen database

SNP	closest gene	Effect allele (A1)	Other allele (A2)	SUA effect (mg/dl)	SUA s.e.	SUA p-value	heart failure effect (mg/dl)	heart failure s.e.	heart failure p-value
rs10214468	SLC17A1	a	t	0.059	0.0084	3.46E-11	0.0412	0.0198	0.0373
rs11599171	SLC16A9	a	g	0.052	0.0066	1.52E-13	-0.0351	0.0119	0.0032
rs11650989	BCAS3	a	g	0.042	0.0071	2.48E-08	0.0059	0.0136	0.6629
rs11689803	GTF3C2	a	t	-0.049	0.0064	4.87E-13	0.0145	0.0122	0.2336
rs11728055	AC006499.1	a	c	0.31	0.01	3.52E-168	-0.0187	0.0240	0.4348
rs11732272	SLC2A9	a	g	0.11	0.0079	5.76E-43	NA	NA	NA
rs11736814	TAPT1-AS1	t	c	-0.16	0.022	5.01E-12	-0.0395	0.0345	0.2513
rs11815391	A1CF	a	g	-0.046	0.0067	1.13E-10	0.0078	0.0130	0.5510
rs12418845	FLRT1	a	g	-0.072	0.012	3.09E-08	0.0030	0.0252	0.9043
rs12647851	TAPT1-AS1	t	c	-0.11	0.016	1.38E-10	-0.0452	0.0441	0.3056
rs13114077	RNA5SP153	t	c	-0.054	0.0072	1.37E-12	0.0190	0.0117	0.1046
rs13115661	CLNK	t	c	-0.14	0.013	1.13E-25	-0.0329	0.0187	0.0791
rs13128385	SLC2A9	c	g	-0.2	0.026	1.24E-13	-0.0382	0.0251	0.1286
rs13132625	SLC2A9	a	c	-0.12	0.013	1.64E-18	-0.0045	0.0224	0.8428
rs1394125	UBE2Q2	a	g	0.043	0.0063	9.78E-11	0.0094	0.0130	0.4682
rs1395	SLC5A6	a	g	0.036	0.0061	2.92E-08	0.0275	0.0124	0.0262
rs1424949	AC009480.4	t	g	-0.034	0.0058	3.99E-08	0.0084	0.0119	0.4799
rs1466462	SIPA1	c	g	0.042	0.0056	1.42E-12	0.0016	0.0114	0.8855
rs17632159	TMEM171	c	g	-0.038	0.0061	2.00E-09	-0.0085	0.0131	0.5144
rs2004659	NUDT17	t	c	0.032	0.0056	3.35E-08	-0.0254	0.0119	0.0330
rs2154219	SLC17A4	a	g	0.076	0.013	3.40E-08	-0.0009	0.0310	0.9779
rs2169612	HSP90AB3P	c	g	0.045	0.0068	4.84E-10	0.0298	0.0134	0.0257

rs2240466	BAZ1B	a	g	-0.052	0.0083	2.98E-09	0.0093	0.0170	0.5843
rs2311597	AC073257.2	a	g	0.035	0.0059	1.59E-08	0.0108	0.0118	0.3594
rs2581824	SFMBT1	a	c	0.047	0.0056	8.35E-16	0.0093	0.0116	0.4210
rs2941454	HNF4G	a	g	0.043	0.0054	7.25E-14	-0.0034	0.0119	0.7732
rs301395	CARMIL1	a	c	0.05	0.0057	7.76E-17	0.0013	0.0123	0.9148
rs3114018	ABCG2	a	c	-0.086	0.0053	3.38E-52	-0.0014	0.0113	0.9042
rs3184504	SH2B3	t	c	0.036	0.0054	2.6E-10	-0.0433	0.0115	0.0002
rs4247633	AP003774.4	t	c	-0.037	0.0054	8.03E-11	0.0156	0.0114	0.1693
rs4401177	BRE	a	g	0.058	0.0093	2.77E-09	-0.0317	0.0184	0.0857
rs4466013	MEPE	a	g	-0.21	0.024	1.6E-16	0.0054	0.0371	0.8853
rs4490426	LNCPRESS2	a	c	-0.049	0.0059	9.01E-15	0.0083	0.0118	0.4813
rs4617927	HLF	t	g	0.034	0.0055	5.8E-09	0.0088	0.0114	0.4382
rs4693946	RP11-10L7.1	t	c	-0.068	0.011	1.99E-09	0.0073	0.0308	0.8122
rs4930556	NRXN2	c	g	-0.063	0.0058	1.89E-24	0.0277	0.0116	0.0168
rs4966014	IGF1R	t	c	-0.044	0.0065	1.68E-10	-0.0252	0.0129	0.0499
rs4971059	TRIM46	a	g	-0.039	0.0062	3.02E-09	-0.0118	0.0114	0.3015
rs6858510	TAPT1-AS1	a	g	0.26	0.03	2.88E-16	-0.0320	0.1245	0.7970
rs729761	RP11-344J7.3	t	g	-0.046	0.0063	3.05E-12	0.0141	0.0124	0.2567
rs732021	CYB5B	c	g	-0.057	0.0096	2.55E-08	0.0237	0.0175	0.1747
rs7436833	TAPT1-AS1	t	c	-0.14	0.011	2.46E-32	-0.0059	0.0284	0.8362
rs7485577	NXPH4	a	g	-0.036	0.0062	2.55E-08	0.0163	0.0126	0.1944
rs7664572	SLC2A9	t	c	-0.12	0.014	4.47E-17	0.0115	0.0320	0.7200
rs7669296	SLC2A9	c	g	-0.16	0.026	9.21E-09	0.0216	0.0466	0.6433
rs814174	TMEM14C	a	g	-0.037	0.0057	1.06E-09	0.0050	0.0118	0.6695

Abbreviations: SNP, single nucleotide polymorphism; SUA, serum uric acid; GWAS, genome-wide association study; s.e.,standard error

Supplement Table 6 MR results for association between serum urate concentrations and coronary artery disease, angina, myocardial infarction

Exposure	Outcome	No.SNP	Methods	OR	95%CI	*P*	Horizontal pleiotropy *P* for Egger intercept	Heterogeneity *P* for Cochran's Q	*P* for MR PRESSO global test
Urate	Coronary artery disease	21	IVW	1.03	0.98-1.08	0.235		0.370	
			WM	1.02	0.96-1.08	0.620			
			MR-Egger	0.99	0.91-1.07	0.715	0.240		
			Simple mode	1.03	0.91-1.15	0.653			
			Weighted mode	1.01	0.95-1.06	0.806			
			MR-PRESSO	1.04	0.98-1.09	0.189			0.274
Urate	Angina	23	IVW	1.00	0.99-1.01	0.414		0.062	
			WM	1.00	0.99-1.01	0.508			
			MR-Egger	1.00	0.99-1.01	0.657	0.886		
			Simple mode	1.00	0.99-1.01	0.200			
			Weighted mode	1.00	0.99-1.01	0.329			
			MR-PRESSO	-	-	-			0.087
Urate	Myocardial infarction	15	IVW	1.00	0.99-1.01	0.744		0.560	
			WM	1.00	0.99-1.01	0.978			
			MR-Egger	1.00	0.99-1.01	0.794	0.632		
			Simple mode	1.00	0.99-1.01	0.715			
			Weighted mode	1.00	0.99-1.01	0.879			
			MR-PRESSO	1.00	0.99-1.01	0.709			0.797

MR, mendelian randomization; IVW, inverse-variance weighted, WM, weighted median; MR-PRESSO, MR pleiotropy residual sum and outlier.

Supplement Figure1: Forest plots of the causal estimates of SUA traits and CVD

A

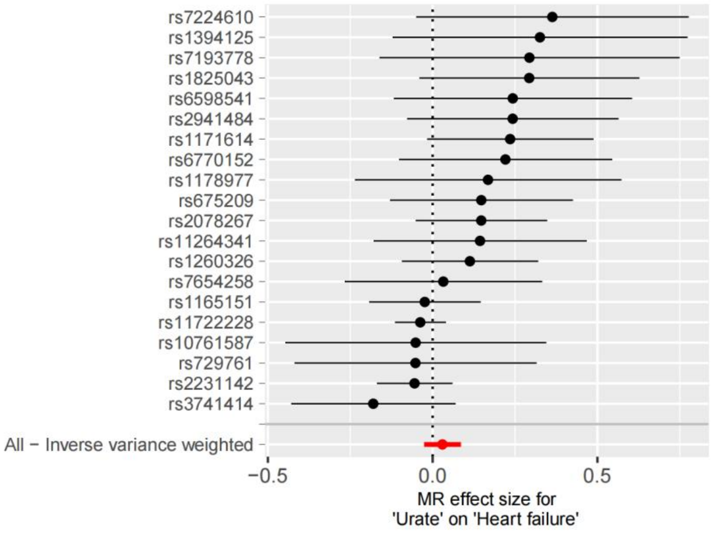

B

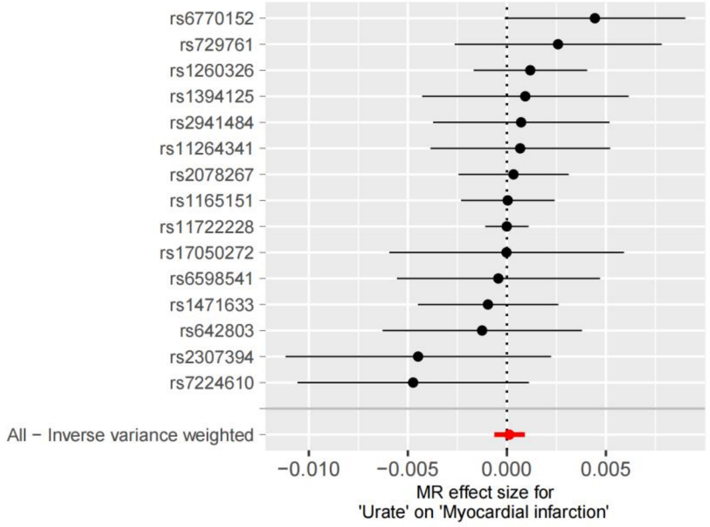

C

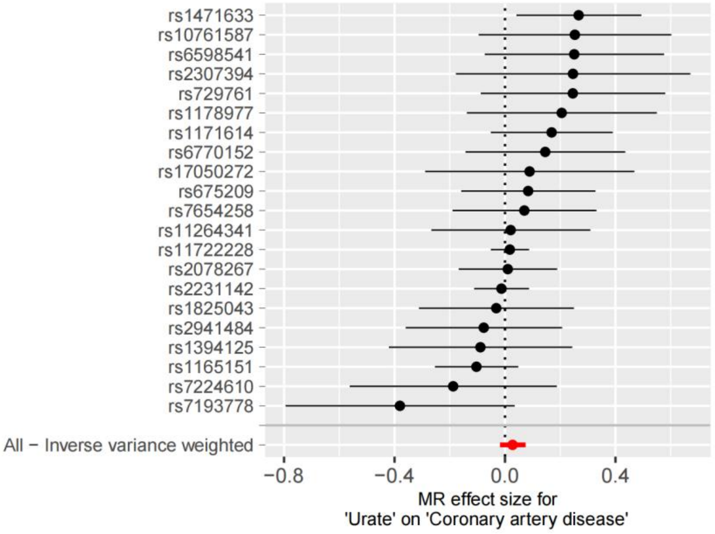

D

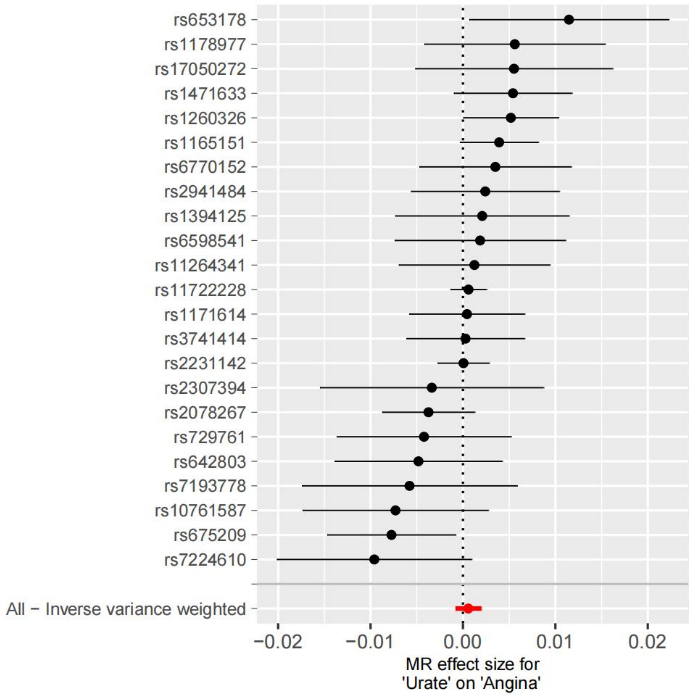

A : Forest plots of the causal estimates of SUA traits and heart failure

B: Forest plots of the causal estimates of SUA traits and myocardial infarction

C: Forest plots of the causal estimates of SUA traits and coronary artery disease

D: Forest plots of the causal estimates of SUA traits and angina

Supplement Figure 2: Scatter plots of the causal estimates of SUA traits and CVD

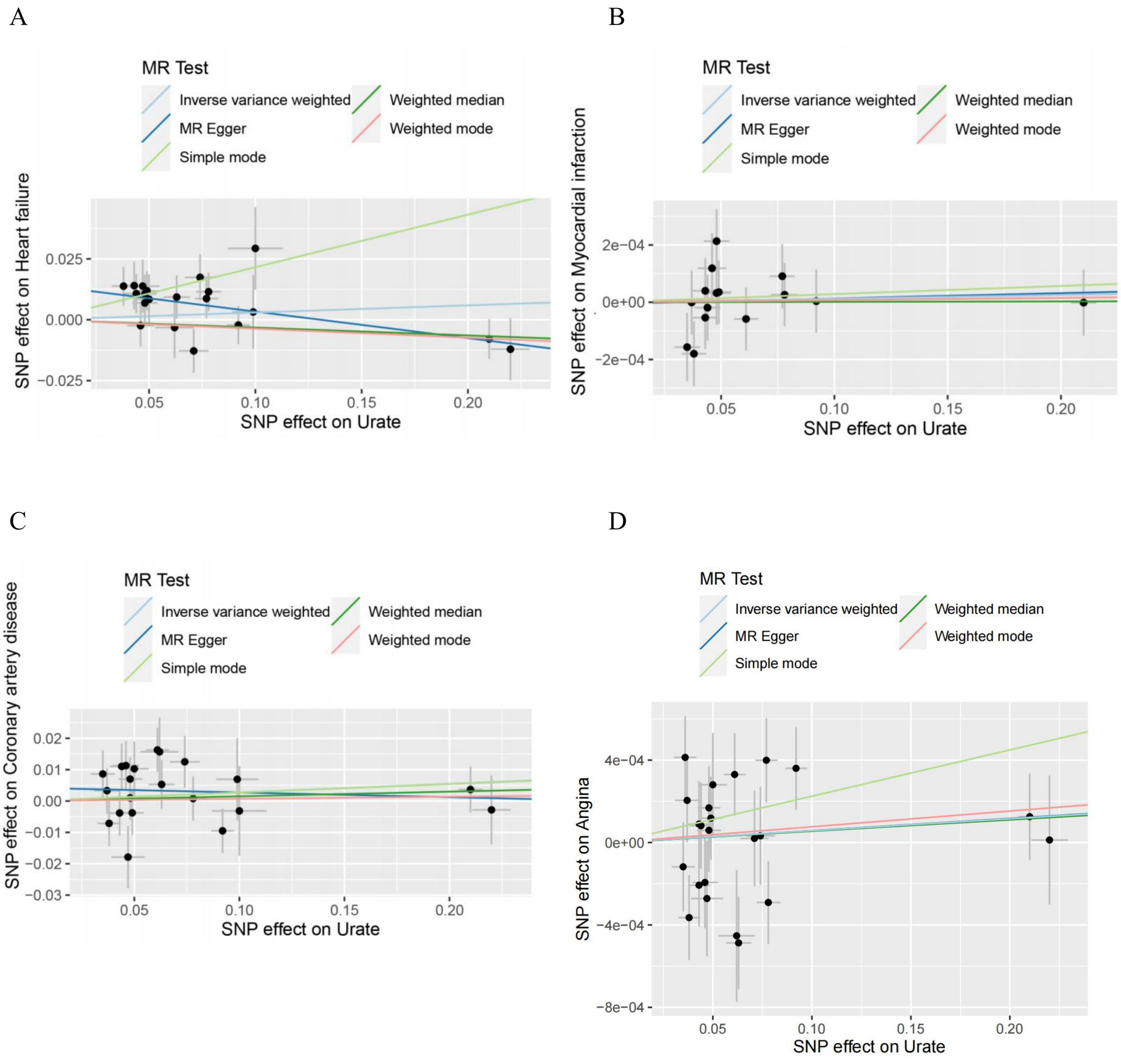

A : Scatter plots of the causal estimates of SUA traits and heart failure

B: Scatter plots of the causal estimates of SUA traits and myocardial infarction

C: Scatter plots of the causal estimates of SUA traits and coronary artery disease

D: Scatter plots of the causal estimates of SUA traits and angina
